# Genetic common variants associated with cerebellar volume and their overlap with mental disorders: a study on 33,265 individuals from the UK-Biobank

**DOI:** 10.1038/s41380-022-01443-8

**Published:** 2022-01-25

**Authors:** Tom Chambers, Valentina Escott-Price, Sophie Legge, Emily Baker, Krish D. Singh, James T. R. Walters, Xavier Caseras, Richard J. L. Anney

**Affiliations:** 1 MRC Centre for Neuropsychiatric Genetics and Genomics, Division of Psychological Medicine and Clinical Neurosciences, School of Medicine, Cardiff University, Cardiff, UK; 2 Cardiff University Brain Research Imaging Centre (CUBRIC), School of Psychology, Cardiff University, Cardiff, UK; 3 UK Dementia Research Institute, Cardiff University, Cardiff, UK

**Keywords:** Neuroscience, Genetics, Psychiatric disorders

## Abstract

Interest in the cerebellum is expanding given evidence of its contributions to cognition and emotion, and dysfunction in various psychopathologies. However, research into its genetic architecture and shared influences with liability for mental disorders is lacking. We conducted a genome-wide association study (GWAS) of total cerebellar volume and underlying cerebellar lobe volumes in 33,265 UK-Biobank participants. Total cerebellar volume was heritable (*h*^2^_SNP_ = 50.6%), showing moderate genetic homogeneity across lobes (*h*^2^_SNP_ from 35.4% to 57.1%; mean genetic correlation between lobes *r*_g_ ≈ 0.44). We identified 33 GWAS signals associated with total cerebellar volume, of which 6 are known to alter protein-coding gene structure, while a further five mapped to genomic regions known to alter cerebellar tissue gene expression. Use of summary data-based Mendelian randomisation further prioritised genes whose change in expression appears to mediate the SNP-trait association. In total, we highlight 21 unique genes of greatest interest for follow-up analyses. Using LD-regression, we report significant genetic correlations between total cerebellar volume and brainstem, pallidum and thalamus volumes. While the same approach did not result in significant correlations with psychiatric phenotypes, we report enrichment of schizophrenia, bipolar disorder and autism spectrum disorder associated signals within total cerebellar GWAS results via conditional and conjunctional-FDR analysis. Via these methods and *GWAS catalogue*, we identify which of our cerebellar genomic regions also associate with psychiatric traits. Our results provide important insights into the common allele architecture of cerebellar volume and its overlap with other brain volumes and psychiatric phenotypes.

## Introduction

The cerebellum has historically been ascribed solely to a role in movement coordination, however, increasing evidence has underlined its relevance in cognition and emotion [1], with expansive functional connectivity with non-motor cortical regions [2–4] and activity during a wide-range of cognitive tasks [5]. Lesions during cerebellar development not only lead to motor alterations but also to cognitive and emotional deficits [6], and represent the second highest risk factor for autism spectrum disorder (ASD) [7]. Cerebellar anatomical alterations have also been identified in most other neurodevelopmental/psychiatric disorders, with particularly strong cerebellar-specific evidence in schizophrenia [8], but also in attention deficit hyperactivity disorder (ADHD) [9] and mood disorders [10], general liability to clinical mental disorders [11] and adolescent psychopathology [12].

Cerebellar volume reductions have also been reported in unaffected relatives of people with schizophrenia, bipolar disorder and depression, being also the only structure commonly reduced across all three disorders [13] and suggesting cerebellar volume reductions to be associated with genetic risk for mental disorders. Indeed, analysing shared altered genetic expression across these three disorders as well as ADHD and ASD shows strong cerebellar tissue enrichment [14].

Twin studies show cerebellar volume to be heritable (*h*^2^ = 33.6–86.4%) [15], but little is known about its polymorphic architecture. In this study, we aim to undertake an in-depth investigation of the common variant influences of total cerebellar volume, their association with altered cerebellar gene expression, genetic overlap with other cortical and subcortical anatomical phenotypes, and importantly, with several of these psychiatric disorders shown to be associated with cerebellar anatomical abnormalities (i.e. ASD, ADHD, schizophrenia, bipolar disorder and depression). While a recent omnibus-GWAS study [16] using an overlapping sample to ours, included 30 cerebellar volume metrics, none of these corresponded to total cerebellar volume and did not explore genetic association with mental disorders.

## Methods

### Total cerebellar volume measure generation

This study utilises T1-weghted structural brain magnetic resonance imaging (MRI) image derived phenotypes (IDPs) data for ~40,000 individuals from UK Biobank (http://www.ukbiobank.ac.uk/) (Supplementary Methods). The generation and semi-automated quality control of these IDPs by UK Biobank has been described previously [17]. Our research group accessed the data in two batches, each containing approximately half of the total sample (henceforth wave 1 and wave 2), which we analysed separately before being meta-analysed.

We generated a summated total cerebellar grey-matter volume measure from the 28 cerebellar lobule IDPs [18], aside from Crus I vermis due to its small size [19]. To explore the reliability of UK Biobank’s cerebellar volume measures, for the 1273 participants in our study who have been scanned twice by UK Biobank within a 5-year interval (with these second scans not included in our main analyses), between-scan intraclass correlation indicated a high test-retest reliability of our cerebellar volume metric (ICC = 0.92). Following outlier removal, we obtained residual total cerebellar volume values after correction for covariates of age, sex, head motion, date of scan and imaging centre attended, and head and table position in the scanner (see Supplementary Methods for details). We scaled these values, with beta values reflecting differences in standard deviations (SD) of residual cerebellar volume.

### Genotyping and quality control

A description of UK-Biobank’s genetic-data collection, quality control and imputation processes can be found elsewhere (http://www.ukbiobank.ac.uk/scientists-3/genetic-data/). We applied additional quality controls independently to each wave’s genotypes (Supplementary Methods), including restriction to unrelated individuals of British/Irish ancestry (>96% sample) (Supplementary Fig. 1). Following local processing, from initial samples of 21,390 and 26,541 participants with genetic-data in wave 1 and wave 2,19,170 and 22,808 participants remained, with 7,003,604 and 6,935,580 genetic markers, respectively.

### Genome-wide association study (GWAS)

After merging genetic and cerebellar volume data, we conducted two separated GWASs using *PLINK* (v1.9) [20] including 17,818 participants in wave 1 (age mean[min,max]= 63[45,80]yrs, 53% female) and 15,447 participants in wave 2 (age mean [min, max] = 65 [48,81] yrs, 53% female) (Supplementary Table 1). The first ten genetic PCs were inputted in these analyses to account for any potential remaining population structure.

### SNP-based heritability (h^2^_SNP_)

Lower-bound estimates of narrow-sense single nucleotide polymorphism (SNP)-based heritability (*h*^2^_SNP_) for each wave were calculated using GCTA-GREML (Genome-wide complex trait analysis—genome-based restricted maximum likelihood) (64 bit; v1.26.0) [21, 22] on the raw genotypes and including covariates of the first 10 genetic principal components.

### Identification of independent GWAS signals

Regional GWAS signals were refined using GCTA-COJO [21, 23] to identify independent index/lead SNPs (Supplementary Methods). Extended-LD regions are provided (*r*^2^ > 0.2 with index SNP and *p* < 0.05 association). LocusZoom [24] was used to visually inspect these signal peaks (Supplementary Fig. 2).

### Comparison of GWASs from wave 1 and wave 2

Several methodologies were deployed to assess similarity between summary statistics from both waves, including between-wave SNP replication, LDSC [25] genetic correlation and *PLINK* [20] polygenic score analyses (Supplementary Methods).

### Meta-analysis

We meta-analysed the two waves’ GWASs using METAL (2011-03-25 release) [26], weighting effect sizes by the inverse of the standard errors and retaining only the 6,193,476 markers present in both waves. Independent index SNP identification and SNP-based heritability estimates were calculated using the same methods as outlined above, creating a merged wave dataset for SNPs’ LD structure and estimates of h^2^_SNP_ (GCTA-GREML*)*.

### Within cerebellum analysis—by lobe analysis

To investigate the homogeneity of cerebellar volume genetic architecture, we ascertained the GCTA-GREML h^2^_SNP_ and LDSC between-lobe genetic correlation estimates for 7 cerebellar lobes based on demarcations of primary, horizontal and posterolateral fissures: anterior (I-V), superior posterior (VI-Crus I), inferior posterior (Crus II-IX) and flocculonodular (X) hemispheres and vermal regions of the latter three (Supplementary Methods).

### Functional annotation and cerebellar gene expression

We physically mapped the extended-LD regions of each index SNP (*r*^2^ > 0.2 to Index SNP) to nearby transcripts and functionally annotated index SNPs and high LD proxy SNPs (*r*^2^ > 0.8 to index SNP) for SNP consequences using several methods (Supplementary Methods). We additionally mapped these proxy SNPs to GTEx-v7 expression quantitative trait loci (cis-eQTL), focusing on directly relevant cerebellar-labelled tissues, but also including analyses in other brain and whole-blood tissues. Use of Summary data-based Mendelian Randomisation (SMR) [27, 28] allowed for assessing mediation via altered cerebellar gene expression of our meta-GWAS identified SNP-cerebellar volume associations, and separation of pleiotropic associations from those caused by linkage within the genomic region (Supplementary Methods).

### Genetic correlation analysis

We used LDSC to estimate genetic correlations between our total cerebellar volume meta-GWAS summary statistics and previously published GWAS summary statistics from two studies that included different sub-regional cerebellar measures [16, 29], cortical and subcortical anatomical measures [30–32], anthropomorphic traits (http://www.nealelab.is/uk-biobank/), and psychiatric disorders of schizophrenia, bipolar, major depression and ASD and ADHD [33–37] (Supplementary Methods). We additionally ascertained genetic overlap between cerebellar volume and these psychiatric disorders, irrespective of direction of effect, using conditional and conjunctional false discovery rate (FDR) analysis [38] (see Supplementary Methods). This included analysis of genetic enrichment in our cerebellar GWAS using stratified quantile–quantile (Q–Q) plots, and investigation of which of our COJO-identified GWAS signals contained SNPs showing evidence for a pleiotropic association with a psychiatric phenotype (conjunctional-FDR < 0.01). Finally, for our COJO proxy SNPs, we inspected *GWAS catalogue* for previous reports of associations with these psychiatric traits, as well as for any additional traits (Supplementary Methods).

## Results

### Between-wave results’ reliability and validity

**Fig. 1:**
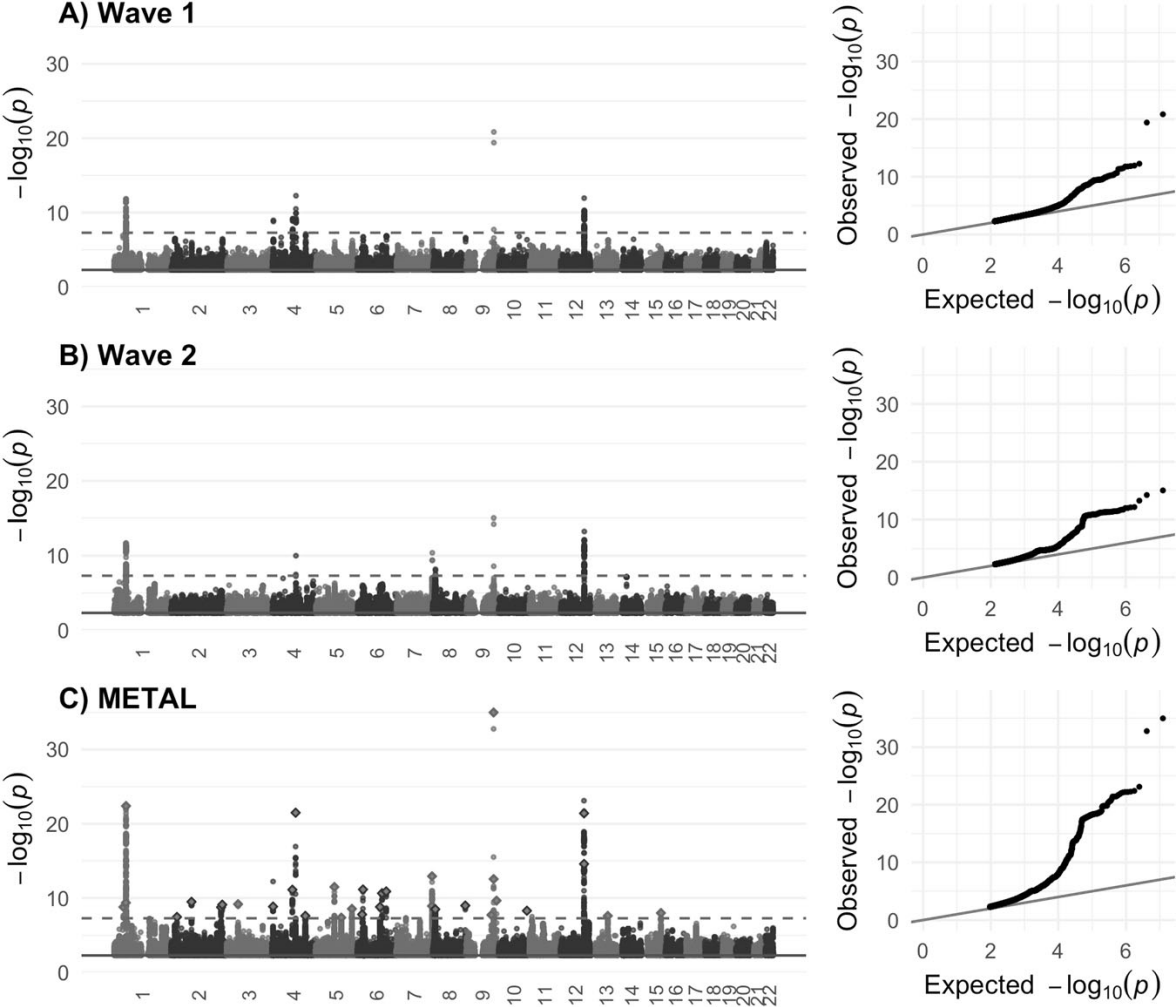
Manhattan and Q-Q plots for each wave GWAS and the meta-GWAS. Manhattan plots of associations with total cerebellar volume for (**A**) wave 1 data release (*n* = 17,818), (**B**) wave 2 data release (*n* = 15,447), and (**C**) wave 1  +  wave 2 combined METAL meta-analysis.For the METAL plot, the 33 COJO-identified independent index SNPs are highlighted (diamond shape). In all cases, the dashed line indicates genome-wide significance at *p* < 5 × 10^−8^. Quantile–quantile (Q–Q) plots for each GWAS are provided next to the Manhattan plot. For all plots, points *p* > 5 × 10^−3^ (solid line) are removed for ease of interpretation.

The GWASs of cerebellar volume identified 6 independent genome-wide significant index SNPs in each wave (Fig. 1; Supplementary Table 2A, B). Each showed high replication in the alternate wave, with all six wave 2 SNPs replicated in wave 1 (*p* < 0.0083{0.05/6}) and all but one wave 1 index SNPs replicated in wave 2. Four were genome-wide significant in both waves. SNP-based heritability estimates were similar across waves (wave 1 h^2^_SNP_[standard error (SE)] = 46.8 [3.4]% and wave 2 h^2^_SNP_ [SE] = 45.3 [3.9]%; lambda GC 1.12 [intercept 1.01] and 1.10 [intercept 1.01], respectively), with a very strong between-wave genetic correlation (*r*_*g*_ [SE] = 1.0 [0.1], *p* = 2.2 × 10^−33^). All polygenic scores derived from one wave significantly predicted total cerebellar volume in the opposing wave, with the most variance explained by wave 1 GWAS derived polygenic scores being at a SNP inclusion p-threshold (*p*_T_) <0.01 (19,210 SNPs, Δ*R*^2^ = 1.9%, *p* = 5.3 × 10^−118^) and at *p*_T_ < 0.1 for wave 2 GWAS derived polygenic scores (146,489 SNPs, Δ*R*^2^ = 1.3%, *p* = 3.9 × 10^−100^) (Supplementary Table 3).

### Meta-analysis of GWAS results for wave 1 and wave 2

Given the high correlation between waves, we combined both waves’ summary statistics in a meta-GWAS (*n* = 33,265, SNPs = 6,193,476) (Fig. 1). The SNP-based heritability estimate in the combined sample was *h*^2^_SNP_ [SE] = 50.6[2.0]% (lambda GC 1.18 [intercept 1.02]).

### Cerebellar lobe analysis

**Fig. 2:**
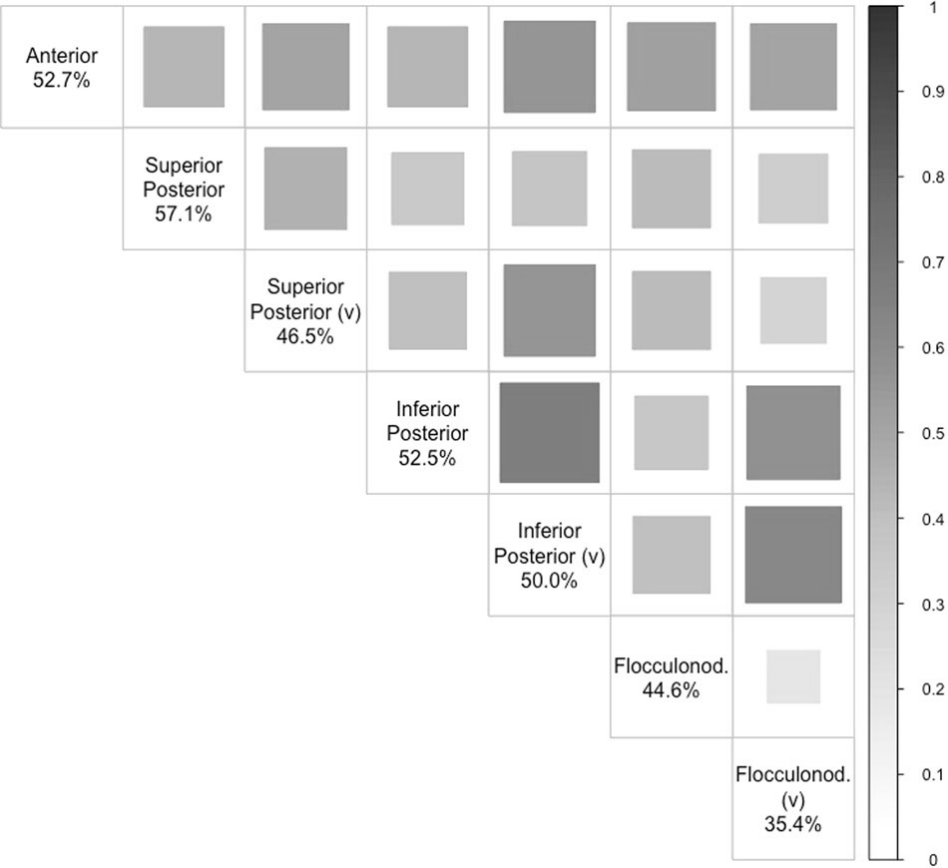
Genetic correlation between the seven cerebellar lobes. Tile size and shade represent genetic correlation values (*r*_g_) between lobes calculated using LDSC regression analysis. Diagonal values of SNP-based heritability estimates calculated using GCTA-GREML. All correlations passed Bonferroni correction *p* < 0.0024{0.05/21}. v vermis.

SNP-based heritability estimates across individual lobes were similar to the overall cerebellar heritability, except for the lower vermal flocculonodular lobe heritability estimate (*h*^2^_SNP_ [SE] = 35.4[1.9]%) (Supplementary Table 4). Between-lobe genetic correlation was moderate for most (between lobes mean *r*_g_ ≈ 0.44) and all survived Bonferroni correction for the number of lobe-pairings tested (*p* < 0.0024 {0.05/21}), being strongest between the inferior posterior hemisphere and vermis (*r*_g_ [95% CI] = 0.66 [0.60, 0.72], *p* = 1.4 × 10^−103^) and weakest between the flocculonodular hemisphere and vermis (*r*_g_ [95% CI] = 0.19 [0.07, 0.30], *p* = 1.3 × 10^−3^) (Fig. 2; Supplementary Table 4).

### Annotation and mapping of genome-wide significant regions from the meta-GWAS

We found 33 conditionally independent index SNPs associated with total cerebellar volume (Table 1; Supplementary Fig. 2). All index SNPs in each wave were present within the 33 meta-GWAS index SNPs, all 33 meta-GWAS index SNPs were at least nominally significant in each wave (*p* values ranging from 7 × 10^−3^ to 1.4 × 10^−21^ for wave 1 and from 5.3 × 10^−3^ to 9.5 × 10^−16^ for wave 2) and with all showing the same direction of effect across waves (Supplementary Table 5).

**Table 1:** Genome-wide association results for total cerebellar volumes in UK Biobank following COJO analysis.

Locus	Cytoband	CHR	Extended-LD range	Index SNP Name	Index SNP Position	A1/A2	Beta_GWAS_ (SE)	P_GWAS_	Beta_COJO_ (SE)	P_COJO_
1	1p34.2	1	40236396..40434968	rs12127002	40384968	A/G	−0.0334 (0.0055)	1.53E−09	−0.0334 (0.0055)	1.36E−09
2	1p32.3	1	50841117..52638689	rs7530673	51558856	A/C	0.0542 (0.0055)	4.00E−23	0.0526 (0.0055)	1.58E−21
2	1p32.3	1	50776624..51682964	rs1278519	50897342	A/C	−0.0344 (0.0055)	4.17E−10	−0.0318 (0.0055)	8.74E−09
3	2p23.3	2	25479624..25619823	rs6546070	25531779	A/G	0.0303 (0.0055)	3.34E−08	0.0303 (0.0055)	4.08E−08
4	2p11.2	2	88749514..89179064	rs7593335	88878133	A/G	0.0345 (0.0055)	3.07E−10	0.0345 (0.0055)	4.22E−10
5	2q35	2	217673928..217980232	rs2542212	217803906	A/G	−0.0331 (0.0055)	1.65E−09	−0.0329 (0.0055)	2.24E−09
6	2q36.1	2	222949007..223309955	rs75779789	223057209	A/G	0.0338 (0.0055)	7.42E−10	0.0336 (0.0055)	1.03E−09
7	3p21.31	3	48184492..50153917	rs7640903	49338465	A/G	0.0339 (0.0055)	6.37E−10	0.0339 (0.0055)	8.62E−10
8	4p16.2	4	4638654..4902425	rs10033073	4775401	A/G	0.0334 (0.0055)	1.34E−09	0.0334 (0.0055)	1.50E−09
9	4q22.1	4	88611354..89316460	rs4148155	89054667	A/G	0.0376 (0.0055)	7.32E−12	0.0376 (0.0055)	9.17E−12
10	4q24	4	102657791..103426409	rs13135092	103198082	A/G	−0.0532 (0.0055)	3.23E−22	−0.0532 (0.0055)	5.57E−22
11	4q31.21	4	145330633..146224823	rs6812830	145613807	A/G	0.0306 (0.0055)	2.39E−08	0.0370 (0.0056)	4.89E−11
12	5q14.2	5	81667102..82008326	rs55803832	81920587	A/C	−0.0383 (0.0055)	3.09E−12	−0.0383 (0.0055)	4.44E−12
13	5q22.2	5	111934537..112311278	rs3846716	112059594	A/G	−0.0302 (0.0055)	4.10E−08	−0.0302 (0.0055)	4.52E−08
14	5q33.3	5	158058006..158536993	rs7380908	158396062	A/C	−0.0326 (0.0055)	2.70E−09	−0.0326 (0.0055)	3.41E−09
15	6p22.3	6	22006131..22184959	rs9393227	22100912	A/G	0.0312 (0.0055)	1.54E−08	0.0314 (0.0055)	1.23E−08
16	6p22.2	6	25264597..28544225	rs1800562	26093141	A/G	−0.0377 (0.0055)	6.75E−12	−0.0379 (0.0055)	5.94E-12
17	6q16.2	6	99654270..100334555	rs546897	100132856	A/G	−0.0332 (0.0055)	1.49E−09	−0.0331 (0.0055)	1.95E−09
18	6q21	6	108635716..109080753	rs1935951	108999101	A/G	0.0368 (0.0055)	2.18E−11	0.0367 (0.0055)	3.06E−11
19	6q22.32	6	126598460..127377494	rs72971190	127088303	A/G	−0.0373 (0.0055)	1.24E−11	−0.0373 (0.0055)	1.46E−11
20	7q36.3	7	156100022..156273180	rs57131976	156167072	A/C	0.0409 (0.0055)	1.12E−13	0.0456 (0.0055)	2.82E−16
20	7q36.3	7	156016471..156178006	rs11764163	156066865	A/G	0.0336 (0.0055)	1.12E−09	0.0391 (0.0055)	2.10E−12
21	8p23.1	8	8042025..11945009	rs2572397	11176403	A/G	−0.0325 (0.0055)	3.11E−09	−0.0325 (0.0055)	4.05E−09
22	8q24.3	8	141983550..142130336	rs6984592	142040038	A/G	0.0335 (0.0055)	9.85E−10	0.0335 (0.0055)	1.35E−09
23	9q31.2	9	109365922..109976563	rs7027172	109571457	A/G	−0.0310 (0.0055)	1.79E−08	−0.0305 (0.0055)	2.78E−08
24	9q33.1	9	119007741..119200439	rs72754248	119061396	A/G	0.0683 (0.0055)	1.08E−35	0.0716 (0.0055)	3.62E−38
24	9q33.1	9	119117887..119553742	rs17220352	119248059	A/G	0.0401 (0.0055)	2.69E−13	0.0455 (0.0055)	2.17E−16
25	9q34.11	9	131364336..132013262	rs3118634	131905854	A/G	−0.0348 (0.0055)	2.14E−10	−0.0348 (0.0055)	2.65E−10
26	10q26.13	10	123306938..123606457	rs4752582	123443605	A/G	−0.0322 (0.0055)	4.89E−09	−0.0322 (0.0055)	5.00E−09
27	12q23.2	12	102349379..102996220	rs5742632	102856474	A/G	−0.0530 (0.0055)	3.90E−22	−0.0482 (0.0055)	5.95E−18
27	12q23.2	12	102405447..103009565	rs703545	102943000	A/G	−0.0437 (0.0055)	2.48E−15	−0.0377 (0.0055)	1.24E−11
28	13q21.33	13	72807523..73006046	rs529059	72933970	A/G	−0.0308 (0.0055)	2.38E−08	−0.0308 (0.0055)	2.42E−08
29	15q25.2	15	82339282..84014925	rs62012045	82521707	A/G	0.0315 (0.0055)	9.79E−09	0.0315 (0.0055)	1.15E−08

*CHR* chromosome, *Extended LD range* *r*^2^ > 0.2 to index SNP and *p* < 0.05 association with cerebellar trait, *β*_*GWAS*_ *(SE)* GWAS original Beta value (Standard Error), *P*_*GWAS*_ GWAS original *p* value, *β*_*COJO*_ *(SE)* Beta value after correcting for neighbouring SNPs (10 Mb sliding window) following GCTA-COJO (Standard Error), *P*_*COJO*_ *p* value following GCTA-COJO.

Functional annotation of the 33 independent GWAS signals (index SNPs and high LD partners *r*^2^ > 0.8) (Supplementary Tables 6, 7A, B) identified 5 containing non-synonymous SNPs leading to altered protein structure. Two of these were flagged as likely deleterious: the missense variants rs1800562 within *HFE* and rs13107325 within *SLC39A8* transcripts. The other three non-synonymous SNPs were flagged as tolerated/benign, being within genes *EIF2AK3*, *PPP2R4 (*alias *PTPA)*, and *MYCL*. A further synonymous annotated SNP located within *PAPPA* gene was within our strongest GWAS signal (rs72754248 Index SNP).

Six of the 33 GWAS signals mapped to genome-wide significant cis-eQTLs in GTEx-v7 cerebellum and cerebellar hemisphere tissue (index SNPs: rs7640903, rs55803832, rs546897, rs2572397, rs6984592 & rs3118634), associating with 14 gene transcripts: *AF131216.5, AMT, CCDC71, GPX1, NCKIPSD, PPP2R4, PTK2, RP1-199J3.5, RP11-247A12.2, RP11-247A12.7, RP11-481A20.10, RP11-481A20.11, VCAN*, and *WDR6* (Supplementary Table 8A, B). When extending analyses to include all brain and whole-blood GTEx-v7 tissues, we found a further 3 GWAS signals mapping to whole-blood eQTLs (*AP3B2* at rs62012045, *CCDC53* at rs5742632 and *REEP5* at rs3846716), moreover the marker rs2572397 revealed additional eQTLs for *ALG1L11P* (Basal Ganglia) and *RP11-981G7.6* (Spinal Cord Cervical C1) (Supplementary Table 8C). SMR analysis found evidence for causal (or pleiotropic) relationships between GWAS and cerebellar gene expression associations for 3 GWAS signals namely at 5q14.2, 8p23.1 and 9q34.11 for 6 transcripts: *PPP2R4*, *RP11-247A12.2*, *RP11-247A12.7, VCAN*, *FAM86B3P* and *FAM85B* (Table 2). The strongest SMR association was observed for *VCAN*, showing a clear relationship between total cerebellar volume GWAS association signals and *VCAN* cerebellar gene expression (Supplementary Fig. 3).

**Table 2:** The number of genes identified by summary data-based Mendelian randomisation (SMR) analysis.

Locus	Cytoband	Tissue	Probe ID	Gene Symbol	Top SMR Marker	Top SMR Marker Position	P (eQTL)	P (GWAS)	P (SMR)	P (HEIDI)	N SNPs HEIDI
12	5q14.2	Cerebellum	ENSG00000038427.11	VCAN	rs55803832	81920587	1.48E−12	3.09E−12	6.93E−07	0.57	10
21	8p23.1	Cerebellum	ENSG00000253893.2	FAM85B	rs2980439	8094870	3.58E−21	1.01E−06	1.40E−05	0.43	20
21	8p23.1	Cerebellar Hemisphere	ENSG00000173295.3	FAM86B3P	rs1878561	8092405	2.85E−19	1.77E−06	2.44E−05	0.39	20
21	8p23.1	Cerebellum	ENSG00000173295.3	FAM86B3P	rs1878561	8092405	2.37E−25	1.77E−06	1.39E−05	0.12	20
25	9q34.11	Cerebellum	ENSG00000119383.15	PPP2R4	rs3118634	131905854	3.99E−16	2.14E−10	5.87E−07	0.27	14
25	9q34.11	Cerebellum	ENSG00000204055.4	RP11-247A12.2	rs3118634	131905854	6.18E−09	2.14E−10	1.87E−05	0.47	13
25	9q34.11	Cerebellar Hemisphere	ENSG00000268707.1	RP11-247A12.7	rs3124505	131887856	1.94E−20	1.31E−08	1.31E−06	0.17	19
25	9q34.11	Cerebellum	ENSG00000268707.1	RP11-247A12.7	rs3118634	131905854	1.16E−20	2.14E−10	1.65E−07	0.23	19

*P (eQTL/GWAS/SMR)* *p* values from the GWAS results, eQTL association, and SMR mediation tests, *P* *(HEIDI)* *p* values from the HEIDI (heterogeneity in dependent instruments) test with *p* > 0.05 indicating pleiotropic (over linkage) associations, *N SNPs HEIDI* number of SNPs used included in the HEIDI test.

### Genetic correlations

We found high positive genetic correlation above Bonferroni-corrected significance threshold (*p* < 0.0014 {0.05/35}) between our total cerebellum meta-GWAS summary statistics and those of previously published regional cerebellar measures (left & right hemispheres; IIV–V, VI–VII and VIII–IX vermal regions [29]: *r*_g_ [95% CI] = 0.91 [0.84, 0.97] and 0.91 [0.84, 0.98]; 0.44 [0.28, 0.60], 0.45 [0.32, 0.57] and 0.56 [0.46, 0.65], respectively; left and right cerebellar regions [16]: *r*_g_ [95% CI] = 0.88 [0.84, 0.93] and 0.99 [0.85, 0.93]; 27 cerebellar lobule regions excluding Crus I vermis [16], *r*_g_ mean [min, max] = 0.65 [0.41, 0.80]) (Supplementary Table 9A). Of the 33 GWAS signals we identified, 28 reached genome-wide significance in these previous works while 5 were novel to the literature (Index SNPs rs6546070, rs6812830, rs3846716, rs3118634 and rs529059). We also found positive genetic correlation (*p* < 0.005 {0.05/10}) between our total cerebellar volume measure and brainstem, pallidum and thalamus volumes, as well as a trend towards a negative correlation with cerebral cortical surface area but which fell short of our Bonferroni-corrected significant threshold (Table 3A). We found no genetic correlations (*p* < 0.0083 {0.05/6}) with any anthropomorphic measure, confirming the results not to simply be reflecting general body size measures (Supplementary Table 9B).

**Table 3:** Genetic correlation of total cerebellar volume with (A) brain-based phenotypes and (B) brain-related phenotypes previously associated with cerebellar anatomy/function.

	*h*^2^_SNP_ (%)	*h*^2^_SNP_ SE (%)	*r*_g_	95% Confidence intervals		*p*	*p*_Bonferroni_
**(A) Brain-based phenotypes**							
Brainstem	31.7	3.4	0.47	0.37	0.58	1.02E−18	1.02E−17
Pallidum	16.9	2.3	0.31	0.19	0.43	0.00000045	0.0000045
Thalamus	16.0	2.1	0.24	0.12	0.36	0.0000645	0.000645
Cortical surface area	35.3	3.2	−0.14	−0.25	−0.04	0.007	0.07
Amygdala	8.4	1.9	−0.18	−0.37	0.01	0.07	0.67
Hippocampus	13.0	2.7	−0.14	−0.29	0.02	0.08	0.84
Caudate	28.6	2.6	−0.07	−0.18	0.04	0.20	1.00
Accumbens	20.2	2.3	−0.07	−0.20	0.06	0.29	1.00
Putamen	28.6	2.8	0.01	−0.10	0.11	0.88	1.00
Cortical thickness	26.5	2.2	−0.01	−0.11	0.10	0.91	1.00
**(B) Brain-related phenotypes**							
Schizophrenia disorder	42.1	1.5	−0.04	−0.10	0.02	0.18	0.90
Bipolar disorder	34.6	1.9	−0.04	−0.12	0.04	0.33	1.00
Attention Deficit Hyperactivity Disorder (ADHD)	22.7	1.7	−0.07	−0.17	0.03	0.18	0.90
Autism spectrum disorder (ASD)	19.5	1.5	−0.10	−0.22	0.02	0.10	0.50
Major Depressive Disorder	7.8	0.5	−0.02	−0.10	0.08	0.61	1.00

*h*^*2*^_*SNP*_ SNP-based heritability estimates (on the observed scale), *SE* standard error, *r*_*g*_ genetic correlation, *p* uncorrected *p* values, *p*_*Bonferronni*_ *p* values adjusted for the number of tests performed regions/traits tested (10 and 6, respectively).

We ascertained the genetic correlation between cerebellar volume and liability to psychiatric diagnoses. None showed significant consistent genetic correlation across the genome with cerebellar volume, even at nominal significance (Table 3B). Stratified Q–Q plots, however, suggested a clear enrichment of schizophrenia signal and, to a less degree, bipolar and ASD associations within our total cerebellar volume variants (Supplementary Fig. 4). No apparent relationship was seen with major depressive disorder or ADHD. Conjunctional-FDR analysis revealed 8 of the 33 GWAS signals showing evidence for a pleiotropic relationship with a psychiatric phenotype (5 with schizophrenia, 2 with bipolar, 1 with ASD, and 1 with ADHD), with one GWAS signal (index SNP rs2572397) associating with more than one psychiatric condition: being with decreased cerebellar volume, decreased schizophrenia and increased ASD risk liability (Supplementary Table 10). In total, the majority (7/9) of pleiotropic associations were in opposing directions of effect to that of cerebellar volume. Finally, we report 2 of our 33 COJO GWAS signals (rs13135092 and rs1935951) as being previously associated with psychiatric traits of schizophrenia, bipolar, ASD, and across- and between-psychiatric disorder diagnoses (Supplementary Table 11A, B).

## Discussion

In this study we examined UK-Biobank brain imaging and genotype data of 33,265 individuals to investigate common allele influences on cerebellar volume. We found total cerebellar volume is moderately heritable in our sample (*h*^2^_SNP_ = 50.6%), identifying 33 independent genome-wide significant signals (index SNPs and SNPs in LD) associated with this trait. We identified 6 within protein-coding sections of the genome while another 5 associated with cerebellar gene expression regulation. We found evidence for pleiotropy of identified variants with schizophrenia, bipolar and ASD. We did not, however, find significant genetic correlations across the whole genome, suggesting a smaller subset of pleiotropic regions and/or opposing direction of effects across these regions.

Our main GWAS of total cerebellar volume identified 33 index SNPs, of which 28 had been reported genome-wide significant (*p* < 5e−8) in previous GWASes of sub-regional cerebellar volume measures [16, 29]. The 5 other index SNPs had previously shown subthreshold associations with some of those sub-regional volumes, while reaching GWAS significance level for our composite total volume measure. This overlap suggests an important genetic homogeneity across cerebellar structures, as previously indicated by cerebellar gene expression research [39], and which is further substantiated by our findings of moderate-to-high genetic correlation between our results and those of previous sub-regional cerebellar GWASes, and also across the 7 cerebellar lobe volumes in which we divided the cerebellum following demarcations of primary, horizontal and posterolateral fissures.

We conducted follow-up analyses of each GWAS signal to identify likely causal SNPs. One signal contained the synonymous SNP rs35565319 in the IGF binging protein protease *PAPPA* transcript, with previous reports of possible cerebellar-specific interactional effects [40], high placenta expression and association with adverse pregnancy outcomes [41, 42] and neuronal survival [43]. Five other GWAS signals contained non-synonymous SNPs altering protein structure. Of the two labelled as likely deleterious, one was the rs13107325 variant within the metal cation symporter *SLC39A8* transcript, being previously associated with a wide-range of traits including inferior posterior and flocculonodular lobule [44], striatum and putamen volumes [44, 45], schizophrenia [33, 45], neurodevelopmental outcomes and intelligence test performance [46, 47] and numerous other factors [44, 48–50] (http://www.nealelab.is/uk-biobank/). The other was the rs1800562 variant (alias Cys282Tyr) within the homoeostatic iron regulator *HFE* transcript, with associations with reduced putamen volume and striatal T2star signal [44], and iron and mineral regulation [44, 51, 52]. The other three non-synonymous SNPs included those within translation initiation factor kinase *(EIF2AK3)*, proto-oncogene transcription factor (*MYCL*) and protein phosphatase 2A activator (*PPP2R4 alias PTPA*) protein-coding regions. The novel *PTPA* finding agrees with previous work of the role of phosphatase 2A controlling cell growth and division, regulating dendritic spine morphology [53] and whose dysfunction is a known cause of spinocerebellar ataxia [54].

We also mapped 6 of GWAS signal regions with cis-eQTLs altering expression of 14 gene transcripts. Expanding the cis-eQTL analysis to additional brain regions and whole blood, we identified a further 3 GWAS signals mapping to 5 cis-eQTLs. SMR further investigated possible cerebellar expression mediation of SNP-trait associations for six gene transcripts at 3 GWAS signal regions, including again the *PPP2R4/PTPA* transcript. The strongest SMR association was with *VCAN*, encoding the extracellular matrix protein Versican, which plays crucial roles in nervous system development [55, 56]. The pseudogenes *FAM86B3P* and *FAM85B* were also identified from the SMR analysis, with *FAM85B* and the other non-coding gene cis-eQTLs for *RP11-481A20.10 and RP11-481A20.11* in the same region having been indicated in mood instability and schizophrenia [57, 58]. While a higher confidence can be placed on SMR identified genes, its requirement for multiple cis-eQTL signals within a genomic region means genes with poorer coverage might be omitted, therefore both cis-eQTL-only and SMR identified genes should be considered for future follow-up work.

In total, therefore, from 732 unique gene transcripts overlapping with the extended-LD regions of our 33 index SNPs, functional annotation and cerebellar tissue gene expression mapping refined this to a list of 21 gene transcripts particularly warranting further interrogation (Supplementary Table 12).

Through inspection of *GWAS Catalogue*, we identified 2 GWAS signals (rs13135092 and rs1935951) previously associated with schizophrenia, and the former also with bipolar disorder, ASD and PGC cross-psychiatric disorder associations. Furthermore, using conjunctional-FDR analysis—leveraging genomic pleiotropy to indicate pleiotropic regions which might be below genome-wide significance for each psychiatric GWAS—we not only confirm psychiatric associations at these 2 GWAS signals with schizophrenia, but also identified 6 other GWAS signals with evidence for psychiatric pleiotropy (rs7530673 and rs1278519 with bipolar disorder; rs7640903 with ADHD; rs3118634 and rs62012045 with schizophrenia; rs2572397 with schizophrenia and ASD). Of these 8 GWAS signals, 6 followed the expected opposing direction of effect as would be predicted from case/control studies [8, 11], e.g. associating with increased psychiatric risk liability and decreased cerebellar size, whereas rs13135092 and rs2572397 showed the same direction of effect for both traits. Related to this, while we found evidence for enrichment of our cerebellar GWAS for schizophrenia, bipolar disorder and ASD using stratified Q–Q plots, in accordance with the majority of other structural brain phenotype GWASs [30, 32], we did not find a whole-genome level correlation when using LDSC, indicating regional heterogeneity of effect directions. These results highlight the benefit of using multiple methods to investigate genetic overlap between traits, as previously stressed [38, 59].

We found strong genetic correlation between our total cerebellar volume GWAS and those of the brainstem, pallidum and thalamus [32] but not other subcortical structures, cortical surface area or thickness [30–32]. These results agree with previous reports of a particular clustering of these three subcortical volumes [32, 60] and contrast to the significant phenotypic correlations amongst most subcortical volumes [32]. Importantly, the gene expression profile of cerebellar grey matter is quite distinct [39]. This shared common architecture, therefore, could be explained by cerebellar white matter connectivity between these regions. The major cerebellar input and output nuclei located within the brainstem and thalamus, respectively. Cerebellar-pallidal interactions are known to occur within the cortex, thalamus and via direct connections [61–63], with joint roles in sensorimotor regulation, learning and reward [61]. The common allele overlap found across these four brain structures, therefore, warrants further research into the neurobiological underpinnings of this potential network and its role in psychopathology, particularly given the association between cerebellothalamic and cerebellar-basal ganglia connectivity dysfunction in individuals with schizophrenia [64, 65].

There are several features of the study design to consider when interpreting the results presented. While the UK Biobank’s homogenous data collection and processing helps decrease methodological variation, the cohort does not represent the general UK population, deviating in important socioeconomic and demographic measures [66]. We further limited our analyses to participants with ancestry similar to a British and Irish reference (>96% sample), limiting the extrapolation of our results to other ancestries. Regarding the imaging data, while visual inspection of each segmentation was not possible due to the cohort size, we believe the UK Biobank’s semi-automated image artefact detection, our removal of outlier measures, confirmation of reliability of cerebellar measures in individuals with repeat scans, and correction for potential noise due to participants’ head motion and position within the scanner improve the validity of our cerebellar measures. The UK Biobank’s IDPs, however, are not optimised for the cerebellum, which can lead to poorer registration and segmentation of individuals lobules [67]. For this reason, as well as the high correlation between lobules and its conserved cytoarchitecture, our main analyses focused on total cerebellar volume. Lack of access to raw genotypes for the psychiatric phenotype GWASs prevented the use of methods such as bivariate GCTA-GREML which could have brought further insight into their genetic relationship with cerebellar volume.

In conclusion, we provide a genome-wide association study of the common genetic variation underlying human cerebellar volume. We find a moderate-to-high heritability for cerebellar volume, with relatively consistent heritability across lobes, and sharing common allele influences with brainstem, pallidal and thalamic volumes. We report enrichment for schizophrenia, bipolar and ASD signals, but not for major depression and ADHD. As a guide for future functional studies, we identify 33 independent index SNPs associated with cerebellar volume and 21 unique candidate genes for follow-up work: 6 protein-coding variants and 14 cerebellar tissue cis-eQTL associations, with 6 (4 common with the latter) showing potential causal relationships with gene expression. Overall, these results advance our knowledge on the common allele architecture of the cerebellum and pave the way to further research into the neurobiological basis of its anatomy, and associations with psychiatric conditions.

## Supplementary information

Supplemental methods

Supplemental Tables

Supplemental Figures

## Data Availability

Summary statistics from all GWAS analyses run are available from *GWAS catalog (GCP000196)*.

## Code availability

All genetic analyses used are open-source. Phenotype creation was performed by UK Biobank. Code for the creation of residual cerebellar values for genetic analyses (R), polygenic score (PLINK), or SNP lookup (R/GWAS catalog API) can be provided on request.
