# Supplementary material for: Genetic common variants associated with cerebellar volume and their overlap with mental disorders: a study on 33,265 individuals from the UK-Biobank
Source: Mol Psychiatry. Author manuscript; Available in PMC 2022 May 26. (PMC9126806; doi:10.1038/s41380-022-01443-8)
Supplement: Supplemental methods [file EMS140784-supplement-Supplemental_methods.docx]

**Supplementary Methods**

*UK Biobank*

UK Biobank (<https://www.ukbiobank.ac.uk>) [1] is a large-scale biomedical database and research resource, globally accessible to those underlying health-related research. Between 2006-2010, UK Biobank recruited 500,000 volunteer participants who provided consent to share detailed health, lifestyle and demographic, biometric and genetic information. An eventual 100,000 individuals will additionally undergo brain imaging in several centres across the UK [2]. Based on successive downloads of UK Biobank data of approximately 20,000 participants each (wave 1: 21,390 individuals with cerebellar volume data; wave 2: 18,301 individuals with cerebellar volume data), we analysed these separately as independent samples (referred to as wave 1 and 2, respectively). UK Biobank acquired informed consent from all subjects. Ethics for the UK Biobank was granted by the North West Multi-Centre Ethics Committee and the data was released to us under project ref 17044. UK Biobank is generously supported by its founding funders the Wellcome Trust and UK Medical Research Council, as well as the Department of Health, Scottish Government, the Northwest Regional Development Agency, British Heart Foundation and Cancer Research UK.

*Total cerebellar volume measure generation*

Due to the difficulty of visually inspecting each of the ~40,000 images individually, as a quality control we removed individuals with missing and outlier values, assuming that extreme values could reflect problems during data processing. We removed individuals missing any of our key covariates (listed below) and individuals with outlier (>5× median absolute deviation from overall median) total cerebellar or total brain grey- and white-matter volume (UK-Biobank data-field code: [25010](https://biobank.ndph.ox.ac.uk/showcase/field.cgi?id=25010)). In a univariate multiple linear regression model we regressed total cerebellar volume on total brain volume, age (UK-Biobank data-field code: [21003-2.0](https://biobank.ctsu.ox.ac.uk/crystal/field.cgi?id=21003)), age^2^ (2^nd^ degree orthogonal polynomial), sex ([31](https://biobank.ctsu.ox.ac.uk/crystal/field.cgi?id=31)), age^2^×sex, mean resting-state functional MRI head motion averaged across space and time points ([25741-2.0](http://biobank.ctsu.ox.ac.uk/crystal/field.cgi?id=25741)) (log transformed; [21001-2.0](https://biobank.ndph.ox.ac.uk/showcase/field.cgi?id=21001)), imaging centre attended ([54-2.0](https://biobank.ctsu.ox.ac.uk/crystal/field.cgi?id=54)), date attended imaging centre ([53-2.0](http://biobank.ctsu.ox.ac.uk/crystal/field.cgi?id=53)), X-, Y- and Z-head position in the scanner ([25756](https://biobank.ctsu.ox.ac.uk/crystal/field.cgi?id=25756), [25757](https://biobank.ctsu.ox.ac.uk/crystal/field.cgi?id=25757), [25758](https://biobank.ctsu.ox.ac.uk/crystal/field.cgi?id=25758)) and starting table-Z position ([25759](https://biobank.ctsu.ox.ac.uk/crystal/field.cgi?id=25759)). These cerebellar residuals showed a normal distribution. We used R(3.6.0) (<https://www.R-project.org/>) for the generation of our phenotype and all statistical analyses.

*Genetic quality control*

We harmonised and applied additional quality control (independently) to each wave’s raw genotypes from the UK-Biobank using the genotypeqc function (<https://github.com/ricanney/stata>). All *Stata* functions described therein leverage *PLINK* (v1.90b5.4; [www.cog-genomics.org/plink/1.9/](http://www.cog-genomics.org/plink/1.9/)) [3], and as has been described previously [4].  Briefly, all markers were harmonised to genome build hg19 and common nomenclature based on the Haplotype Reference Consortium r1.1. We excluded markers based on imputation quality score (<0.8), individual marker missingness (>2%), low minor allele count (MAC<5; and a minor allele frequency (MAF) < 0.1% of total 500k UK Biobank sample), deviations from Hardy-Weinberg equilibrium (p< 1×10^–10^) and the deviations from the expected MAF (MAF; >4 standard deviations (SD) from GBR MAF reported in 1000G phase 3). Individuals were removed with excess overall marker missingness rate (>2%) or heterozygosity (>4 × SD from sample mean), those of non-British/Irish ancestry (defined as >4 × SD from 1000G phase 3 GBR sample mean based on first 3 principal components (PCs)) and those with close relatives in the cohort (estimated kinship coefficient > 0.0442 i.e. 3^rd^ degree relatives, coefficient of relatedness > 12.5%). For wave 2 this included removing individuals with close relatives in wave 1.

### *Identification of independent GWAS signals*

Regional GWAS signals were refined to identify independently associated index/lead SNPs by applying a stepwise conditional analysis using the COJO (multi-SNP-based conditional & joint association analysis using GWAS summary data) function in GCTA (64bit; v1.93.2beta) [5, 6], using SNP LD structures from each wave’s data.

*Comparison of GWASs from* wave *1 and* wave *2*

Firstly, two-sided binomial sign tests assessed the replication of each wave’s COJO identified index SNPs in the other wave (p< 0.05/number of index SNPs identified). Secondly, genetic correlation (rg) between the waves was assessed using the LDSC software (1.0.1) [7], by regressing SNP associations (products of the z-scores between the two traits) on their linkage disequilibrium (LD) scores (using the supplied pre-computed 1000G EUR LD scores). All summary statistics were limited to a common subset of HapMap3 SNPs prior to analysis. Thirdly, polygenic scores were generated using *PLINK* for all participants in each wave, using the summary statistics from the other wave (clumping r^2^> 0.2), filtering SNPs at 10 different p-value thresholds: p< 0.5, 0.1, 0.05, 0.01, 0.001, 1×10^-4^, 1×10^-5^, 1×10^-6^, 1×10^-7^ & 1×10^-8^ and repeating this with and without including regions of long-range LD as defined from 1000G phase 3 EUR. Multiple linear regression was used to ascertain the unique variance of total cerebellar volume explained by each polygenic score (ΔR^2^), accounting for the same covariates as used to generate the GWAS (see above section) and calculated by subtracting the R^2^ of the model without covariates from the R^2^ of model with covariates. Bonferroni correction was applied for the number of polygenic score tests performed (p< 0.0013{0.05/(10×2×2)}).

### *Within cerebellum analysis – by lobe analysis*

We divided the cerebellum into lobes based on demarcations of primary, horizontal and posterolateral fissures as outlined previously [8], though grouping hemisphere volumes and separating the flocculonodular lobe. This created 7 lobes, being hemispheres of the anterior (I-V), superior posterior (VI-Crus I), inferior posterior (Crus II-IX) and flocculonodular (X) and separate vermal regions of the latter three (excluding the Crus I vermis). The same quality control procedures, regression on covariates, GWAS analyses for each wave (wave 1: 17,813; wave 2: 15,438; total: 33,251), meta-analysis and GCTA-GREML h^2^_SNP_ estimation were conducted as outlined above and in the main body. We also provide the LDSC estimate of h^2^_SNP_ for each lobe, calculated by regressing SNP’s trait association (χ^2^) on their LD. We also ascertained the LDSC estimate of between lobe genetic correlations (replacing χ^2^ with the product of z-scores from the two lobe measures), with Bonferroni adjusted p-values (significance threshold p_Bonferroni_< 0.05) provided following correction for the number of tests (p< 0.00024 {0.05/21}). Note that we were unable to exclusively use GREML or utilise the bivariate GREML genetic correlation routine as only GWAS summary statistics were available for non-UK Biobank GWAS.

### *Functional annotation and cerebellar gene expression*

Physical annotation of transcripts (<ftp://ftp.ensembl.org/pub/grch37/current/gtf/homo_sapiens/Homo_sapiens.GRCh37.87.gtf.gz>) was applied using overlap of each index SNP’s extended LD-range (SNPs r^2^>0.2 to index SNP and p<0.05 GWAS association) with transcripts boundaries. Index and “proxy” high LD-partner SNPs (r^2^>0.8 and within 500kb of index SNP, using 1000G GBR phase-3 LD reference) were functionally annotated with SNP consequence (<http://www.ensembl.org/>), combined annotation-dependent depletion (CADD) Phred-like scores [9], Polyphen category [10] and SIFT category [11].

These index and proxy SNPs were also mapped to expression quantitative trait loci (eQTL) of GTEx-v7 transcript data from cerebellum and cerebellar hemisphere labelled tissues ([https://gtexportal.org/home](https://gtexportal.org/home/)). There is also potential gain in power by not limiting to just these tissues of interest [12]. Therefore, we expanded analysis to all 13 GTEx-v7 brain (cerebellum, cerebellar hemisphere, amygdala, anterior cingulate cortex, caudate nucleus, cortex, frontal cortex, hippocampus, hypothalamus, nucleus accumbens, putamen, spinal cord and substantia nigra) and whole blood tissues data.

Summary data-based Mendelian randomization (SMR) (<https://cnsgenomics.com/software/smr>; v.103) [13, 14] was used to further explore whether genome-wide signals were mediated by altered cerebellar gene expression. To exclude possibly causal/pleiotropic relationships from those caused by linkage, the HEIDI (heterogeneity in dependent instruments) was used to detect heterogeneity of associations within a region (p_HEIDI_≥ 0.05 indicates not due to linkage) for transcripts where a minimum of 10 SNPs were available in the model. We applied a SMR-wide Bonferroni correction based on the number of transcripts that passed inclusion criteria, for both the cerebellum (p_SMR_< 1.42×10^-6^{0.05/3526}) and cerebellar hemisphere (p_SMR_< 2.09×10^-5^{0.05/2389}) labelled tissues.

*Genetic correlation - Downloaded summary statistics*

While no previous study includes a total brain volume analysis, regional structural cerebellar measures have been included in previous brain-wide GWASs which have included data from earlier releases of UK Biobank. These were from Zhao et al (2019) [15] (ANTs (<http://stnava.github.io/ANTs/>) defined left & right cerebellar hemispheres and 3 vermal divisions; n= 19,629 EUR) and Smith et al (2021) [16] (FreeSurfer [17] defined left & right cerebellum and FSL FAST [18] defined 28 individual cerebellar lobules; n = 33,224 EUR). Of note, for cerebellar results, we also assessed the number of our index SNPs identified in our total cerebellar volume meta-GWAS not present in the previously published regional cerebellar results; deeming novel SNPs as those where their extended LD region at least 500kb away from any previously identified index SNP provided in each study’s supplementary tables (or in the case of Smith et al, where index SNPs refer to phenotype clusters, taken from their GWAS summary statistics), and with no previously identified index SNP within r^2^> 0.1 of our index SNP (using 1000G p3 GBR LD reference).

Brain-based measures were those from the ENIGMA group for mean total cortical thickness and surface area using FreeSurfer analysis (n= 33,992 EUR) [19], and for the hippocampus (n= 26,814 EUR) [20] and other subcortical volumes of the putamen, pallidum, thalamus, amygdala, nucleus accumbens, caudate nucleus and brainstem (n= 37,741 EUR) [21].

For brain-related psychiatric and neurological traits, we used the latest GWAS summary statistics for schizophrenia (40,675 cases; 64,643 controls) [22], bipolar disorder (20,352 cases; 31,585 controls) [23], major depressive disorder (59,851 cases; 113,154 controls) [24], autism spectrum disorder (ASD) (18,381 cases; 27,969 controls) (ASD) [25] and attention deficit hyperactivity disorder symptom scores (ADHD) (17,666 children) [26].

As several of the identified variants were associated with anthropomorphic measures, in a post-hoc analysis we wished to ascertain that the identified cerebellar variants were independent from a collection of anthropomorphic measures collected from the full UK-Biobank cohort (<http://www.nealelab.is/uk-biobank/> GWAS round 1 2017 release version limited to EUR ancestry). These included standing height (data-field: [50](https://biobank.ndph.ox.ac.uk/showcase/field.cgi?id=50); n= 336,474), sitting height ([20015](https://biobank.ndph.ox.ac.uk/showcase/field.cgi?id=20015); n= 336,172), birth weight ([20022](https://biobank.ndph.ox.ac.uk/showcase/field.cgi?id=20022); n= 193,063), body mass index ([21001](https://biobank.ndph.ox.ac.uk/showcase/field.cgi?id=21001); n= 336,107), weight ([21002](https://biobank.ndph.ox.ac.uk/showcase/field.cgi?id=21002); n= 336,227) and body fat percentage ([23099](https://biobank.ndph.ox.ac.uk/showcase/field.cgi?id=23099); n= 331,117).

All downloaded summary statistics were harmonised to genome build hg19 and common nomenclature based on the Haplotype Reference Consortium r1.1 and underwent the same procedural steps as outlined above (including HapMap3 filtering). In addition to LDSC estimates of genetic correlation (rg), we also report the LDSC estimated SNP-based heritability scores for each trait on the observed scale. Of note, methods (e.g. LD reference used, calculations on the observed or liability scale) and results for trait heritability estimation might differ to those respective original studies, therefore, please see the respective original papers. Bonferroni correction was used for each set of correlations (cerebellar traits: p< 0.0014{0.05/35}; brain-based traits: p< 0.0050{0.05/10}; anthropomorphic traits: p< 0.0083{0.05/6}; and brain-related traits: p< 0.0083{0.05/6}).

*Conditional and conjunctional false discovery rate (FDR)*

In addition to using genetic correlation analyses to ascertain if a similar direction of SNPs’ effects were seen across the genome for total cerebellar volume and the aforementioned psychiatric traits of interest (schizophrenia, bipolar disorder, major depressive disorder, ASD & ADHD), we used Conditional and Conjunctional false discovery rate (FDR) analyses to identify any global and regional genomic pleiotropy with these traits, irrespective of direction. The methods for these approaches and their advantages in improving identification of shared genetic architecture and susceptibility loci between traits have been well-described previously [27, 28] (<https://github.com/precimed/pleiofdr>). These analyses included various prior quality and data reduction steps as outlined in the online methods (<https://github.com/precimed/pleiofdr>) *(Matlab version 2020a v5)* and we used the default settings described therein (aside from our use of a more extensive long-range of high LD reduction <https://genome.sph.umich.edu/wiki/Regions_of_high_linkage_disequilibrium_(LD)> [29]). This included correcting for genomic inflation (leveraging intergenic SNPs) so as to control for spurious enrichment due to population stratification or cryptic relatedness and random pruning (r^2^<0.1) of SNPs (1000G phase3 EUR reference panel).

Following these data preparation stages, we produced stratified quantile-quantile (Q-Q) plots. Single-trait Q-Q plots are used to compare the GWAS p-values to their empirical distributions (both -log_10_); with enrichment of statistical associations indicated as a leftward deviation from the diagonal of the null hypothesis. Stratified Q-Q plots build on this single-trait Q-Q plot by stratifying SNPs by their strength of association with a second trait (at increasing association strengths of p<1×10^-1^, p<1×10^-2^, p<1×10^-3^), being each of the psychiatric traits used in the above genetic correlation analyses. Overall genetic enrichment for a psychiatric trait is indicated by successive leftward deviations from the diagonal null hypothesis line at increasing associator strength with the psychiatric trait.

In addition to investigating general enrichment of signal, we used Conditional and Conjunctional analyses to investigate which of our COJO-identified independent genome-wide significant signals contained SNPs showing a pleiotropic association with a psychiatric trait. Conditional FDR extends empirical Bayesian false discovery rate methods [30], utilising the pleiotropy common across the human genome to condition one trait’s associations (e.g. cerebellar volume) on another related trait (e.g. each psychiatric trait) and, therefore, increase the power to identify true associations of the first trait. Each conditional FDR value, therefore, reflects the probability the SNP’s effect is null for total cerebellar volume given its total cerebellar volume and psychiatric trait p-values of association are as small or smaller than those observed (i.e. cerebellar association | psychiatric trait association). This was then repeated in the opposing direction, conditioning each psychiatric trait associations on their total cerebellar volume associations (i.e. psychiatric trait association | cerebellar association). For each of our COJO-identified independent GWAS signals for cerebellar volume, we then obtained the conjunctional FDR value for each SNP within the extended LD region (r2>0.2 to Index SNP, p<0.05 association with total cerebellar volume), being the maximum conditional FDR of these two conditioned analyses (i.e. max(cerebellum | psychiatric , psychiatric | cerebellum) ) and providing a conservative estimate for the SNP FDR of association with both traits (again, irrespective of direction). We highlighted regional GWAS signals containing SNPs with conjunctional FDR < 0.01 {FDR of 0.05/5 psychiatric traits}. Such analysis, therefore, can indicate SNPs and regions with evidence for pleiotropic association, which might have been missed if limiting to analyses only of genome-wide significant associations in both traits and provide additional information to the other approaches used such as whole genome genetic correlation and regional *GWAS catalog* analyses (discussed below).

*GWAS catalog*

Finally, we searched in *GWAS Catalog* for previously identified associations of each of our COJO index SNPs and high LD proxy SNPs (r2>0.8 and <500kb to Index SNP, 1000G GBR p3) using the *R* *gwasrappidd* package to interface with *GWAS Catalog REST* API [31] (*ENSEMBLE* build 103, *dbSNP* 154, build GRCh38.p13). We provide results for all traits showing a genome-wide significant (p < 5×10^-8^) association, as well as another supplementary table specifically highlighting those traits with particular psychiatric relevance.

**References**

1. Collins R. What makes UK Biobank special? Lancet. 2012;379:1173–1174.

2. Littlejohns TJ, Holliday J, Gibson LM, Garratt S, Oesingmann N, Alfaro-Almagro F, et al. The UK Biobank imaging enhancement of 100,000 participants: rationale, data collection, management and future directions. Nat Commun. 2020;11:2624.

3. Chang CC, Chow CC, Tellier LC, Vattikuti S, Purcell SM, Lee JJ. Second-generation PLINK: Rising to the challenge of larger and richer datasets. Gigascience. 2015;4:7.

4. Underwood JFG, Kendall KM, Berrett J, Lewis C, Anney R, Van Den Bree MBM, et al. Autism spectrum disorder diagnosis in adults: Phenotype and genotype findings from a clinically derived cohort. Br J Psychiatry. 2019;215:647–653.

5. Yang J, Lee SH, Goddard ME, Visscher PM. GCTA: A tool for genome-wide complex trait analysis. Am J Hum Genet. 2011;88:76–82.

6. Yang J, Ferreira T, Morris AP, Medland SE, Madden PAF, Heath AC, et al. Conditional and joint multiple-SNP analysis of GWAS summary statistics identifies additional variants influencing complex traits. Nat Genet. 2012;44:369–375.

7. Bulik-Sullivan B, Finucane HK, Anttila V, Gusev A, Day FR, Loh PR, et al. An atlas of genetic correlations across human diseases and traits. Nat Genet. 2015;47:1236–1241.

8. Tiemeier H, Lenroot RK, Greenstein DK, Tran L, Pierson R, Giedd JN. Cerebellum development during childhood and adolescence: A longitudinal morphometric MRI study. Neuroimage. 2010;49:63–70.

9. Kircher M, Witten DM, Jain P, O’roak BJ, Cooper GM, Shendure J. A general framework for estimating the relative pathogenicity of human genetic variants. Nat Genet. 2014;46:310–315.

10. Adzhubei IA, Schmidt S, Peshkin L, Ramensky VE, Gerasimova A, Bork P, et al. A method and server for predicting damaging missense mutations. Nat Methods. 2010;7:248–249.

11. Kumar P, Henikoff S, Ng PC. Predicting the effects of coding non-synonymous variants on protein function using the SIFT algorithm. Nat Protoc. 2009;4:1073–1081.

12. Qi T, Wu Y, Zeng J, Zhang F, Xue A, Jiang L, et al. Identifying gene targets for brain-related traits using transcriptomic and methylomic data from blood. Nat Commun. 2018;9:2282.

13. Zhu Z, Zhang F, Hu H, Bakshi A, Robinson MR, Powell JE, et al. Integration of summary data from GWAS and eQTL studies predicts complex trait gene targets. Nat Genet. 2016;48:481–487.

14. Pavlides JMW, Zhu Z, Gratten J, McRae AF, Wray NR, Yang J. Predicting gene targets from integrative analyses of summary data from GWAS and eQTL studies for 28 human complex traits. Genome Med. 2016;8:84.

15. Zhao B, Luo T, Li T, Li Y, Zhang J, Shan Y, et al. Genome-wide association analysis of 19,629 individuals identifies variants influencing regional brain volumes and refines their genetic co-architecture with cognitive and mental health traits. Nat Genet. 2019;51:1637–1644.

16. Smith SM, Douaud G, Chen W, Hanayik T, Alfaro-Almagro F, Sharp K, et al. An expanded set of genome-wide association studies of brain imaging phenotypes in UK Biobank. Nat Neurosci. 2021;24:737–745.

17. Dale AM, Fischl B, Sereno MI. Cortical surface-based analysis: I. Segmentation and surface reconstruction. Neuroimage. 1999;9:179–194.

18. Zhang Y, Brady M, Smith S. Segmentation of brain MR images through a hidden Markov random field model and the expectation-maximization algorithm. IEEE Trans Med Imaging. 2001;20:45–57.

19. Grasby KL, Jahanshad N, Painter JN, Colodro-Conde L, Bralten J, Hibar DP, et al. The genetic architecture of the human cerebral cortex. Science (80- ). 2020;367:eaay6690.

20. Hibar DP, Adams HHH, Jahanshad N, Chauhan G, Stein JL, Hofer E, et al. Novel genetic loci associated with hippocampal volume. Nat Commun. 2017;8:1–12.

21. Satizabal CL, Adams HHH, Hibar DP, White CC, Knol MJ, Stein JL, et al. Genetic architecture of subcortical brain structures in 38,851 individuals. Nat Genet. 2019;51:1624–1636.

22. Pardiñas AF, Holmans P, Pocklington AJ, Escott-Price V, Ripke S, Carrera N, et al. Common schizophrenia alleles are enriched in mutation-intolerant genes and in regions under strong background selection. Nat Genet. 2018;50:381–389.

23. Stahl EA, Breen G, Forstner AJ, McQuillin A, Ripke S, Trubetskoy V, et al. Genome-wide association study identifies 30 loci associated with bipolar disorder. Nat Genet. 2019;51:793–803.

24. Wray NR, Ripke S, Mattheisen M, Trzaskowski M, Byrne EM, Abdellaoui A, et al. Genome-wide association analyses identify 44 risk variants and refine the genetic architecture of major depression. Nat Genet. 2018;50:668–681.

25. Grove J, Ripke S, Als TD, Mattheisen M, Walters RK, Won H, et al. Identification of common genetic risk variants for autism spectrum disorder. Nat Genet. 2019;51:431–444.

26. Middeldorp CM, Hammerschlag AR, Ouwens KG, Groen-Blokhuis MM, St. Pourcain B, Greven CU, et al. A Genome-Wide Association Meta-Analysis of Attention-Deficit/Hyperactivity Disorder Symptoms in Population-Based Pediatric Cohorts. J Am Acad Child Adolesc Psychiatry. 2016;55:896-905.e6.

27. Andreassen OA, Djurovic S, Thompson WK, Schork AJ, Kendler KS, O’Donovan MC, et al. Improved detection of common variants associated with schizophrenia by leveraging pleiotropy with cardiovascular-disease risk factors. Am J Hum Genet. 2013;92:197–209.

28. Smeland OB, Frei O, Shadrin A, O’Connell K, Fan CC, Bahrami S, et al. Discovery of shared genomic loci using the conditional false discovery rate approach. Hum Genet. 2020;139:85–94.

29. Price AL, Weale ME, Patterson N, Myers SR, Need AC, Shianna K V., et al. Long-Range LD Can Confound Genome Scans in Admixed Populations. Am J Hum Genet. 2008;83:132–135.

30. Efron B, Tibshirani R. Empirical bayes methods and false discovery rates for microarrays. Genet Epidemiol. 2002;23:70–86.

31. Magno R, Maia A-T. gwasrapidd: an R package to query, download and wrangle GWAS catalog data. Bioinformatics. 2020;36:649–650.
