# Supplementary material for: Genetic common variants associated with cerebellar volume and their overlap with mental disorders: a study on 33,265 individuals from the UK-Biobank
Source: Mol Psychiatry. 2022 Jan 25;27(4):2282–90. doi: 10.1038/s41380-022-01443-8 (PMC9126806; doi:10.1038/s41380-022-01443-8)
Supplement: Supplementary file 1 [file 41380_2022_1443_MOESM3_ESM.docx]

**All**

**GBR**

**All**

**GBR**

**B) Phase 2**

**A) Phase 1**

**Supplementary Figure 1: Principal component analysis (PCA) of genetic ancestry to British/Irish (GBR) similarity for A) phase 1 and B) phase 2.** PCA was used on the genotypes to identify principal components (PCs). The first 3 PC’s were compared to 1000G phase 3 super-population ancestries using *bim2ancestry* function ([https://github.com/ricanney/stata](https://github.com/ricanney/stata)). Each plot shows the test participant ancestries (yellow squares) and the 1000G super-populations (circles, colour-coded by super-population ancestries). For “All” plots, all test participants are included, while “GBR” plots show the exclusion of those with ancestries dissimilar to British/Irish super-populations (<4 standard deviations).

**Supplementary Figure 2: LocusZoom plots of 33 COJO-identified index SNPs for total cerebellar volume association.** For each identified region, the figure displays the SNP effect p-values from the meta-GWAS on total bran volume (top plot, dots), linkage disequilibrium values between SNPs (top plot, colours), recombination rate from HapMap (top plot, vertical lines) and gene transcripts present in that region (bottom plot). The highlighted red diamonds reflect the index (i.e. lead) SNP identified in each analysis. 

I. **rs12127002**

II. **rs1278519**

III. **rs7530673**

IV. **rs6546070**

V. **rs7593335**

VI. **rs2542212**

VII. **rs75779789**

VIII. **rs7640903**

IX. **rs10033073**

X. **rs4148155**

XI. **rs13135092**

XII. **rs12500946**

XIII. **rs6812830**

XIV. **rs55803832**

XV. **rs3846716**

XVI. **rs7380908**

XVII. **rs9393227**

XVIII. **rs1800562**

XIX. **rs546897**

XX. **rs1935951**

XXI. **rs72971190**

XXII. **rs11764163**

XXIII. **rs57131976**

XXIV. **rs2572397**

XXV. **rs6984592**

XXVI. **rs7027172**

XXVII. **rs72754248**

XXVIII. **rs17220352**

XXIX. **rs3118634**

XXX. **rs4752582**

XXXI. **rs703545**

XXXII. **rs529059**

XXXIII. **rs62012045**

**Supplementary Figure 3: Prioritisation of** ***VCAN*** **gene transcript using SMR analysis.** For the identified GWAS signal, we show an association between SNP effect p-values from the meta-GWAS on total bran volume (top) and SNP effects on *VCAN* eQTL expression in GTEx-v7 cerebellum labelled tissue (middle) for all SNPs present in each summary data, in addition to gene transcripts present in that region (bottom). The highlighted red diamonds reflect the top SNP identified in each analysis which, in this instance, was the identified GWAS index SNP rs55803832.

**Supplementary Figure 4: Conditional QQ plots of genetic overlap of total cerebellar volume (tCB) with psychiatric traits of interest.**  Plots display total cerebellar volume meta-GWAS results conditioned on different strengths of association (p_Psych_) with schizophrenia (SCZ), bipolar disorder (BD), major depressive disorder (MDD), autism spectrum disorder (ASD) and attention deficit hyperactivity disorder (ADHD) traits.
